# Improving antibiotic prescribing – Recommendations for funding and pricing policies to enhance use of point-of-care tests

**DOI:** 10.1016/j.hpopen.2024.100129

**Published:** 2024-09-28

**Authors:** Sabine Vogler, Caroline Steigenberger, Friederike Windisch

**Affiliations:** a WHO Collaborating Centre for Pharmaceutical Pricing and Reimbursement Policies, Pharmacoeconomics Department, Gesundheit Österreich GmbH (GÖG/Austrian National Public Health Institute), Stubenring 6, 1010 Vienna, Austria; b Department of Health Care Management, Technische Universität Berlin, Straße des 17. Juni 135, 10623 Berlin, Germany; c Institute of Public Health, Medical Decision Making and Health Technology Assessment, Department of Public Health, Health Services Research and Health Technology Assessment, UMIT TIROL - University for Health Sciences and Health Technology, Eduard-Wallnoefer-Zentrum 1, 6060 Hall in Tirol, Austria; d Department of Management, Institute for Public Management and Governance, Vienna University of Economics and Business, Welthandelsplatz 1, 1020 Vienna, Austria

**Keywords:** Funding, Pricing, Diagnostic, Antibiotic prescribing, Community-acquired acute respiratory tract infections, Point-of-care test

## Abstract

**Introduction:**

Diagnostics can contribute to the improved quality of antibiotic prescribing. However, there is potential to enhance the use of point-of-care tests (POCTs) in general practice. This paper presents fit-for-purpose policy recommendations related to funding and pricing for POCTs applied for community-acquired acute respiratory tract infections (CA-ARTIs).

**Methods:**

The development of the recommendations was informed by an analysis of the current status of funding and pricing policy frameworks for CA-ARTI POCTs in European countries, and an identification of barriers and facilitators for their uptake. Draft recommendations were developed and subsequently revised based on written and verbal feedback from meetings with experts.

**Results:**

The proposal includes four recommendations for policy interventions related to funding and three recommendations regarding pricing policies. Two of the funding policy-related recommendations concern physicians’ remuneration, and two relate to product-specific reimbursement (public coverage) of the CA-ARTI POCTs. The pricing-related recommendations include a proposal to explore the introduction of price regulation, to pilot subscription-fee procurement models and to enhance more strategic approaches in public procurement of CA-ARTI POCTs.

**Conclusions:**

Optimised pricing and funding policies could make a difference in enhancing uptake of CA-ARTI POCTs. It is crucial for the successful implementation of policies to consider country settings. Additionally, supportive policy action is recommended, including the systematic use of health technology assessment, stakeholder communication, and monitoring and evaluation.

## Highlights

• Diagnostics can address antimicrobial resistance by improving antibiotic prescribing.
• Funding and pricing policies can enhance the uptake of point-of-care tests.
• Future-proof policies may require major changes in the health systems.
• Careful implementation considering the country context is important.

## Graphical abstract

**Figure:**
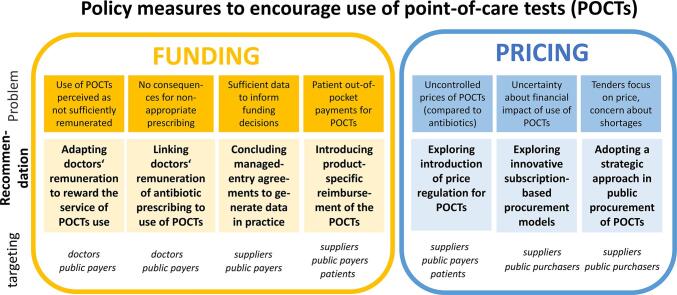

## 1 Introduction

Antimicrobial resistance (AMR) is currently one of the most urgent public health threats [1], [2]. It has been causing and is expected to continue causing millions of deaths, medical complications, and diseases [3], [4], [5]. Further to its health burden, AMR is associated with major economic impacts [6], [7].

Inappropriate prescribing of antibiotics is a major cause of AMR [8]. To address this issue, capacity-building approaches have been undertaken in several countries globally to inform and guide physicians towards more appropriate prescribing [9], [10], [11], [12], [13]. Additional educational and awareness-raising activities are targeted at other health professionals and patients to promote a more responsible use of antibiotics [14].

The quality of antibiotic prescribing can be improved by using a diagnostic which determines if an infection has been caused by a bacterium or a virus [15]. Rapid point-of-care tests (POCTs), which are used by physicians in their practice, provide the requested information within a short time, since there is no need to wait for results from a laboratory, and they reduce immediate antibiotic prescribing [16]. However, some uncertainty about the robustness of the results of some POCTs was reported [17]. While POCT devices have demonstrated acceptable analytical performance in the laboratory setting, the accuracy and precision of the tests was found to be more variable when used at the point of care [18]. Data on the costs of the POCTs for the health systems are frequently not available for several countries. If available, they vary but the costs per patient usually amount to less than € 12. However, the price of the POCT may be higher than the price for an antibiotic and thus some studies concluded that POCTs would not always be cost-effective compared to antibiotic prescribing [19], [20].

Overall, physicians appear to be hesitant to use POCTs. Reported reasons for this include physicians’ confidence in their prescribing decisions, lack of time, and the (perceived) patient expectation to obtain an antibiotic prescription [21], [22], [23]. To address these constraints, actions targeting physicians’ beliefs (educational activities), changes in the working environment, and regulatory provisions may be possible solutions. An increased level of regulation of antibiotics, as well as of good governance in a society, were shown to be associated to lower antibiotic consumption across countries [24], [25].

As is the case for any health technology, the uptake of POCTs will also be influenced by funding and pricing policies [26]. While pricing policies are typically aimed at achieving competitive and affordable prices, funding policies aim to guarantee financial protection for patients and sustainability of health systems. Additionally, funding and pricing policies can be designed with a view to offering incentives to suppliers, health providers and patients so that they manufacture, distribute, prescribe, use, or ask for certain products. A vast body of evidence highlights the contributions of funding and pricing policies for health technologies in achieving defined policy objectives, such as patient access, cost-containment or encouraging innovation; however, most studies relate to medicines [27], [28], [29], [30], [31]. It has also been shown that the methodological design of policies is essential [32], [33], [34] and that certain policies are particularly appropriate for specific health technologies. For instance, specific funding policies, coupled with demand-side measures, have proven effective to enhance the uptake of off-patent medicines such as generic and biosimilar medicines [35], [36].

Given the challenges in ensuring equitable and affordable patient access to therapies with high price tags, several stakeholders have been urging “thinking-out-of-the-box” to encourage development, pilots and implementation of innovative, future-proof policy solutions [37], [38]. In the area of AMR, for instance, there have been discussions around the optimisation of pharmaceutical pricing, procurement, and reimbursement policies, to incentivize the production and marketing of novel antibiotics [39], [40]. A proposal for an action plan for diagnostics in infectious diseases for the United States also stressed the importance of financing and coverage policies [41].

Considering the evidence for other health technologies about the relevance of funding and pricing policies, it can be hypothesized that funding and pricing policies can also provide leverage for the uptake of POCTs. However, for diagnostics, these policies have not been on the political agenda in European countries. At the same time, implementation levels of funding and pricing policies for diagnostics, including POCTs, are overall low [42]. A qualitative study from the United Kingdom pointed to the absence of a funding and reimbursement policy for POCTs as a major barrier to their adaption [43], and a review of barriers and facilitators for the uptake of medical devices in European countries also identified funding issues as one of the barriers [44].

To address this gap, the article aims to shed some light on this neglected area of policy development and implementation for POCTs applied for community-acquired acute respiratory tract infections (CA-ARTI). These infections include influenza, upper respiratory tract infections (e.g., bronchitis and pneumonia), and lower respiratory tract infections (e.g., tonsillitis, pharyngitis, laryngitis, sinusitis, otitis media, certain influenza types, and the common cold), and their treatment has been associated with high levels of inappropriate use of antibiotics [45]. This paper aims to provide guidance to policy-makers about possible avenues for action by presenting fit-for-purpose policy recommendations on funding and pricing of CA-ARTI POCTs, with the purpose to enhancing their uptake, and thus contribute to improved antibiotic prescribing and eventually reductions in AMR. In line with “out-of-the-box” thinking, we deliberately include recommendations of innovative policies which would require major changes in the current European health care systems. Since this “silent pandemic” [5] will continue having major impacts on health, well-being and the economy, we feel a need for considering novel approaches.

## 2 Methods and framework

### 2.1 Setting

The recommendations were developed in the context of the VALUE-Dx project, which aimed to transform medical practice to achieve more evidence-based antibiotic prescribing based on use of cost-effective diagnostics in the community care settings to combat AMR [46].

Specifications on the studied infections (i.e., CA-ARTIs), and the intervention (i.e., POCTs used by physicians in outpatient care, in particular by general practitioners), had been pre-defined before the start of the project.

### 2.2 Health systems and target group

The recommendations were developed for European countries which are advanced in their progress towards Universal Health Coverage and tend to offer a wide range of publicly funded health services. Since the design of how health systems are organised and funded varies across Europe (e.g., private doctors’ practices with a contract with the social health insurance versus employed physicians in primary care units in countries with a national health service (NHS)), the recommendations consider the limited transferability of some policy measures and will specify their applicability for defined health care settings.

While the policy actions aim to encourage the uptake of CA-ARTI POCTs, the recommendations address (national) policy-makers, since they are responsible for planning, designing, introducing, monitoring and, if needed, adapting policies so that they are effective in encouraging physicians to base their antibiotic prescribing on a POCT, when needed.

### 2.3 Conceptional framework for funding and pricing policies

The policy recommendations address the *peri*-launch phase, which is the time span between the regulatory approval for bringing a diagnostic into the market (CE marking) and its actual marketing. Policies are defined as instruments, tools and approaches that allow policy-makers to achieve defined objectives [47], such as to enhance the use of POCTs by physicians. Peri-launch policies for health technologies include funding and pricing policies (see the relevant conceptional frameworks for medicines developed by the World Health Organization (WHO) [48] and the European Observatory for Health Policies and Systems [32], and for diagnostics by Vogler and Windisch 2022 [42]).

The above-mentioned policy framework for diagnostics [42] acknowledges that funding by a third-party payer, such as a national health service or social health insurance, consists of two components: The first concerns payments to the supplier (e.g., the manufacturer or the retailer), which aim to reward the development, manufacturing, launch, and distribution of the diagnostic. This aspect is referred to as product-specific reimbursement. The second funding component is to remunerate for the use of the diagnostic and includes paying the provider (i.e., health professional such as a physician) for the application of the diagnostic (e.g., conducting the diagnostic test, logistical services, maintaining equipment).

Pricing is defined as an action by a government authority to set the price of a medicine or medical device and/or indirectly influence it (e.g., through pricing policies) as well as to monitor, review and possibly adapt it [47]. Linked to pricing, there is public procurement which relates to all aspects surrounding the process of purchasing health technologies by a contracting authority, such as a body of public law (e.g., governments, local health authorities), from economic operators chosen by the contracting authority [49]. Public procurement may be considered as one specific pricing policy [27].

The development of the recommendations was guided by the above-mentioned policy framework and definitions for *peri*-launch policies.

### 2.4 Inputs to inform the drafting of the recommendations

**Fig. 1:**
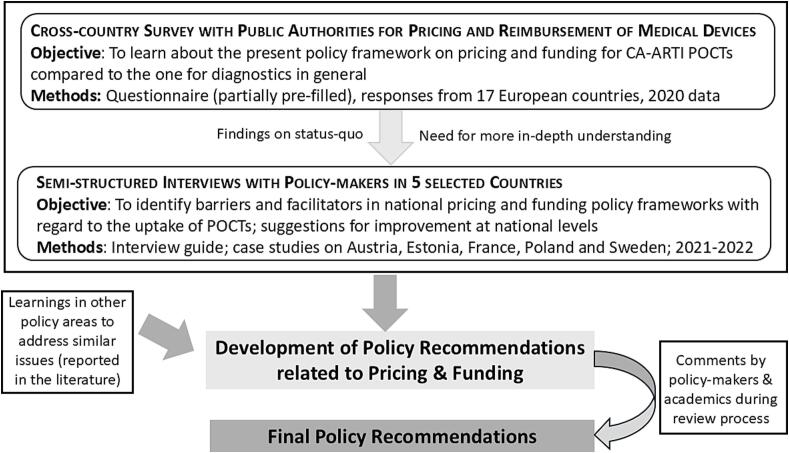
Process for the development of the policy recommendations.

We developed the draft recommendations based on preceding research in which we had studied funding and pricing policies for CA-ARTI POCTs and conducted an analysis of barriers and facilitators in funding and pricing that may influence the uptake of these diagnostics (see also Fig. 1 for a visualisation).

Given an identified lack of data in the literature, information on existing funding and pricing policies was largely collected through a survey with public authorities responsible for pricing and reimbursement, who are members of the Pharmaceutical Pricing and Reimbursement Information Subgroup on Medical Devices (PPRI MD). The Pharmaceutical Pricing and Reimbursement Information (PPRI) network was originally established in 2005 to support competent authorities responsible for pricing and reimbursement of medicines in an exchange of experiences about policy implementation and cross-country learnings [50]. Starting as a network of authorities in European Union (EU) countries, it has grown and includes non-EU member states as well countries from other regions of the world (a total of 50 countries [51]). Public authorities that appreciated the PPRI benefits encouraged the establishment of the Subgroup on Medical Devices in 2018 to offer a similar platform for expert networking on policy questions around medical devices [52]. Members of the PPRI MD Subgroup are technical experts of national competent authorities responsible for pricing and reimbursement working on, or with an interest in, medical devices. In 2020, we invited the members of the PPRI MD Subgroup to respond to a written questionnaire on pricing and funding policies for CA-ARTI POCTs in particular and for diagnostics in general. Among others, it was asked whether price regulation was in place for the studied diagnostics, and if yes, which pricing policies and which criteria were applied. Furthermore, it was surveyed whether the diagnostics were reimbursed (i.e., covered by public funding, and thus no co-payments for the patients) and how doctors were remunerated for the service of using a POCT. The questionnaire surveyed the process around the decision on pricing and funding as well as the use of health technology assessment (HTA) to support these decisions. Aiming for broad coverage, 32 European countries (all 27 Member States, the European Free Trade Association (EFTA) countries Iceland, Norway and Switzerland, the United Kingdom and Turkey were approached. For 22 countries the authors and their colleagues involved in this research prefilled some information obtained in previous research in the questionnaire to ease the workload for the respondents. The survey resulted in the collection of information from 17 European countries. The findings of this mapping exercise were published elsewhere [42].

The status-quo descriptions in the Results section (first columns in Table 1 and Table 2) are based on this mapping exercise across Europe. The collection of the cross-country policy information was a first step and allowed us to study in more detail national policy frameworks and their facilitating and hindering factors on the use of CA-ARTI POCTs. To conduct a more detailed analysis, we chose a case study approach. We selected five countries (Austria, Estonia, France, Poland, and Sweden) as case study countries, and so the sample included countries of different size, different economic status and different levels of centralisation in the organisation of health care. Data was mainly collected through semi-structured interviews with national policy-makers from these countries, primarily drawing from experts of the PPRI MD Sub-group, supplemented by a targeted review of country information. Based on the findings of the cross-country survey and search in country-specific grey literature, peer-reviewed literature and legal documents (also in national languages), we produced a graphical overview of the national pricing and funding system for diagnostics in the study countries, which constituted the first section of the interview guide. The idea was to clarify with the interviewees whether we have captured the national policy framework correctly, and based on a common understanding explored barriers and facilitators in the national policy framework as a second part of the interviews. Thirdly, the interviewees were also invited to provide suggestions for change in the policy environment.

**Table 1:** Recommendations for policy interventions in the area of funding to enhance use of CA-ARTI POCTs by outpatient physicians.

**Current situation and rationale for change**	**Recommendation**	**Applicability**	**Prerequisites and actions to consider in planning, designing and implementing the policy intervention**
In most European countries, physicians are usually not explicitly funded for the service of using a CA-ARTI POCT. In the case of fee-for-service remuneration for a CA-ARTI POCT (in place in a few countries), current tariff schemes may, however, not be perceived as appropriately designed to incentivise the use of CA-ARTI POCTs, because further investments (e.g., trained staff, equipment) are needed.	**RF1: Physicians’ remuneration for the service of using a CA-ARTI POCT** It is recommended to explore applying a tariff scheme for physicians in outpatient practice which remunerates them for the service of using a CA-ARTI POCT in a way that reflects relevant cost components incurred.	This measure is typically applicable in countries in which a remuneration scheme for physicians based on fees-for-service tariffs is in place, which could be adapted. Not applicable in health systems where physicians are employed in primary health care units of the national, regional or local health service.	Identify which cost components (beyond the price of a POCT) are incurred (e.g., staff, logistics) by physicians when using the test. Survey and assess the extent of these costs, consider establishing a reporting system to routinely collect and monitor the data (ideally accompanied by regular interaction between payers and physicians to discuss potential changes in the cost structure). Reorganise the tariff scheme by considering remunerating further cost components in line with the willingness-to-pay (be aware of the increase in public spending). Implement major changes in the tariff scheme on a pilot basis, to allow early learnings and adaptions. Regularly evaluate the tariff scheme and adapt it if needed. Ensure that there are no unintended incentives which would lead to an overuse of POCT: This implies clear guidance on prescribing and use of the test provided by the health system, including the payers, to the prescribers. This guidance would define the situations in which doctors are encouraged and/or obliged to use the POCT, and the situations in which the service of the application of the POCT is remunerated. This guidance needs to be accompanied by robust reporting and documentation (for prescribers) and monitoring systems (managed by the health system), which are to be designed in a manner to keep administrative burden for both doctors and payers as low as possible.
As a rule, physicians, who prescribe an antibiotic inappropriately (even in cases when a CA-ARTI POCT has been recommended or mandated to substantiate their decision), do not face any financial consequences. The rationale of this policy intervention is to enhance use of CA-ARTI POCTs before antibiotic prescribing through the threat of financial consequences for unjustified antibiotic prescribing.	**RF2: Physicians’ remuneration for antibiotic prescribing depending on use of CA-ARTI POCTs** It is recommended to explore an adjustment of the tariff scheme for outpatient physicians, by linking their remuneration for antibiotic prescribing to preceding use of a CA-ARTI POCT in defined cases according to guidelines.	Same for RF2: Applicable in health systems with fee-for-service remuneration for physicians, and not applicable in NHS based systems with employed doctors. Feasibility is also dependant on the health system’s ability to allow implementation of cross-sectorial policy options (see implementation requirements in the next column)	Develop and introduce guidelines that define the conditions for the use of a POCT before antibiotic prescribing. Define clear and transparent rules, including specified exemptions for physicians when antibiotic prescribing is possible and allowed without previous use of a POCT. Develop rules for documentation for the physicians, which offers a lean documentation system that does not impose high administrative burden. Invest in timely information for and dialogue with physicians, especially before the introduction of this policy, to meet possible concerns raised by the physicians towards this measure. Introduce accompanying capacity-building and communication measures for physicians (e.g., on how to communicate the need for the use of the POCTs to patients). This policy requires a legislative and organisational policy framework for cross-sectorial funding which allows linking financial consequences (payment or non-payment in one area – medicines) to the use of another health technology (medical devices area).
Further data, e.g., on clinical utility and validity in practice, are needed, after the CA-ARTI POCT has been made available in the markets. Funding and pricing decisions are frequently made under uncertainty when data are missing.	**RF3: Managed-entry agreements linked to continuous data generation** It is recommended to explore use of managed-entry agreements to link funding of the CA-ARTI POCTs to continuous data generation (e.g., Coverage with Evidence Development, CED) under well-defined conditions.	In principle applicable in both NHS based and social health insurance systems. A legal framework to allow conclusion of managed-entry agreement is required.	Define which indicators are meaningful (a few well-selected data may be sufficient), e.g. on effectiveness, accuracy, user-friendliness and other quality aspects of the tests. Specify who will be responsible for data collection. Ensure that the administrative burden is not getting too large (e.g., through a well-defined set). It is acknowledged that from an organisational point of view data collection in community care may be more difficult than in hospital settings. Ensure transparency in processes and outcomes (e.g., by making the data publicly available, for third party use). Ensure that collected data are actually used in the evaluations to inform funding and pricing decisions. Invest in early dialogue with targeted stakeholders to ensure their cooperation (or at least mitigate any objections). Those who will collect the data should be involved at the stage of the definition of specifications for the data collection and be consulted on possible challenges. Given the novelty of this measure, ideally launch a pilot, and ensure regular evaluations in routine practice.
Reimbursement of a CA-ARTI POCT would financially support patients, as they do not have pay the (full) cost out-of-pocket. Only in some European countries, POCTs are eligible for inclusion into a positive list.	**RF4: Product-specific reimbursement** It is recommended to consider the implementation of product-specific reimbursement for CA-ARTI POCTs to encourage their use.	Typically applicable in health systems with defined benefits package schemes for certain health technologies and services, whose costs are, at least partially, covered by third party payers (frequently public payers). This is mainly the case in social health insurance systems. Not applicable in health settings where POCTs are provided for free in health care facilities (usually NHS countries).	Implement a legal and organisational policy framework for an outpatient benefits package scheme (i.e., a reimbursement list) for medical devices and allow inclusion of those CA-ARTI POCTs, which meet defined eligibility criteria (such as in terms of effectiveness and quality). Define the conditions and criteria under which CA-ARTI POCTs are eligible for the reimbursement list. Publish the criteria. Adapt the process of POCTs inclusion into reimbursement, or, if no reimbursement process for devices is in place, define the parameters of this process to be installed (e.g., establishment of a reimbursement committee). Ensure that reimbursement decisions are based on systematic use of HTA. HTA reports should be published. Ensure re-assessments at regular intervals (systematic monitoring). Develop a disinvestment strategy to allow for the exclusion of those CA-ARTI POCTs that no longer meet the eligibility criteria.

Abbreviations: CA-ARTI: Community-acquired acute respiratory tract infection, HTA: Health Technology Assessment, NHS: National Health Service, POCT: Point-of-care test, RF: Recommendation related to funding.

**Table 2:** Recommendations for policy interventions in the area of pricing to enhance use of CA-ARTI POCTs by outpatient physicians.

**Current situation and rationale for change**	**Recommendation**	**Applicability**	**Prerequisites and actions to consider in planning, designing and implementing the policy intervention**
CA-ARTI POCTs may not be competitive compared to antibiotics with assumingly lower prices. Thus, prices of POCTs may constitute a barrier to access and use.	**RP1: Price regulation to achieve affordable prices** It is recommended to explore the introduction of price regulation, to make CA-ARTI POCTs more affordable and competitive.	All health systems where no price regulation for POCTs is yet in place (at the time of writing, applicable for all European countries).	Implement price regulation based on the most appropriate pricing policy for CA-ARTI POCTs, with a view to avoiding or mitigating unintended effects. Suitable pricing policies include internal price referencing (i.e. aligning the price of additional POCTs to the other existing POCTs) and price capping, with consideration of value-based elements for new POCTs (i.e. granting higher prices for those POCTs which meet defined criteria in terms of effectiveness, accuracy, etc.). The latter pricing decisions could be informed by an HTA. Balance the trade-off between affordability (lower, more competitive prices) and availability (markets losing attractiveness). Develop an implementation plan, which comprises components such as stakeholder dialogue and further strategies to address potential opposition of targeted stakeholder groups (e.g., suppliers). Consider launching a pilot, ensure evaluation and adaption (and extension) of the policy if needed.
Increased uptake of publicly funded CA-ARTI POCTs may challenge public budgets but there is uncertainty about the extent of the financial impact. Novel procurement models are needed that are able to deal with this unclarity and offer some predictability and planning security for both public procurers as well as suppliers.	**RP2: Subscription-based procurement models** It is recommended to explore innovative procurement policies, including subscription-based procurement (“Netflix” model), in which defined volumes are independent from per unit prices.	In principle applicable to all health systems, however the systems should have the capacity to introduce and manage this policy option, as it requires strategic planning, intensive stakeholder negotiations (with manufacturers) and dialogue (with prescribers) and strong forecasting and monitoring systems.	Balance the trade-off between returns perceived as low by suppliers and potential over-use of POCTs. Establish the procurement arrangements based on accurate forecasts, as far as possible. Nominate an institution to be in charge of managing this contract. Introduce accompanying measures (e.g., a managed-entry agreement, HTA). Inform suppliers in advance (e.g., through consultation processes). Inform prescribers about the mechanisms of the system and encourage them to use the POCTs, where recommended (clear guidance). Given the novelty of this measure, it is recommended to first launch a pilot, which will be assessed based on a clear evaluation plan. Take in consideration lessons learned from similar procurement models (e.g., experimented for medicines).
Public procurement focused on driving down prices may risk discouraging suppliers to bid. There is the concern that this may result in availability issues (withdrawal of suppliers from markets, shortages).	**RP3: Strategic procurement** It is recommended to adopt a strategic approach to public procurement which aligns preparation, launch of calls, assessment, and award of bids to defined objectives. Moving towards more strategic procurement includes exploring the use of additional award criteria beyond the price, pooled procurement, market research, tenders awarded to multiple bidders, and strategies to mitigate possible shortages.	In principle applicable to all health systems, and of particular relevance to those that currently procure POCTs.	Consider further award criteria beyond the price (such as security of supply, quality), as also stipulated in the “Most Economically Advantageous Tender” (MEAT) criteria concept in the EU public procurement legislation. Explore approaches of pooling volumes and capacity, to offer larger (and more attractive) markets and to build capacity (e.g., centralised procurement intra-country, cross-country tendering). Award a contract to multiple bidders (e.g., division of the market among bidders through defined quota). Decide strategically on the contract duration (i.e., managing the trade-off between competition in shorter-term contracts and security of supply), in combination with other elements of strategic procurement. Use supportive tools and instruments (e.g., HTA, managed-entry agreements). Know the market (by conducting market research where needed, dialogue with suppliers). Define key performance indicators and monitor them. Select the procurement practices and techniques which are most appropriate for CA-ARTI POCTs. Use e-procurement. Apply transparent and clear procedures.

Abbreviations: CA-ARTI: Community-acquired acute respiratory tract infection, HTA: Health Technology Assessment, POCT: Point-of-care test, RP: Recommendation related to pricing.

The expert interviews (one each per case study country) were held virtually between December 2021 and May 2022. The interview guideline was shared in advance with the interviewee, together with the informed consent form. We obtained informed consent before the interviews. We decided not to record the interviews, since this may be hindering to create an atmosphere of trust in which the interviewees could freely express their ideas. As a rule, two researchers of the authors’ team participated in the interviews, one of them taking the lead in asking the questions and the other one taking notes. Key points of the interviews were summarised in minutes, which were shared with the interviewees for validation. The information gained on barriers and facilitators and suggestions for improvements obtained in the interviews was further worked on in a qualitative content analysis: We applied a deductive–inductive approach to identify barriers, facilitators and levers for change with a view to transferability, i.e. those that could be applicable to further health systems beyond the studied ones (the findings on the barriers and facilitators analysis was published elsewhere [53]).

### 2.5 Development of the draft recommendations and review processes

The research findings guided us in drafting the policy recommendations, which aim to respond to identified gaps in the funding and pricing policy framework and to address misalignment between the intention of policies and their effects (e.g., policies apparently not reaching stated objectives, unwanted effects). In addition to the learnings from the case study countries, we considered the findings on barriers, facilitators, challenges and good practice examples for diagnostics identified in earlier stages of this project. We also transferred, where appropriate, learnings from the pharmaceutical sector, which has a more advanced funding and pricing policy framework, to the diagnostics area, thus drawing from the literature. We were guided by the “thinking-out-of-the-box” principle: the current situation of limited implementation of funding and pricing policies for diagnostics in the European countries, aggravated by a perceived lack of a policy-supportive environment (e.g., in terms of the institutional framework, public funding, or capacity), did not prevent us from proposing novel policy measures.

The review process of the draft policy recommendations lasted from September to November 2022. To allow for broad representation of the reviewers’ community, we presented the draft recommendations in three expert meetings and invited participants to comment verbally. In addition, the technical document presenting the draft policy recommendations was shared with the participants of the review meetings, who were granted two to three weeks for written review. The recommendations were presented at an onsite Value-Dx consortium meeting, which was attended by researchers from academia and applied science (medicine, biology, economics, information science) and the diagnostics industry, at a virtual meeting of the multi-stakeholder Expert Advisory Panel of the Value-Dx project, that involved selected representatives from research and policy-making with knowledge and interest in POCTs, and at a webinar that we organised for policy-makers to gain their perspective. The latter was attended by representatives of the PPRI network, as described above; some of them had provided information on policies in their country in the preceding survey and/or participated in the interviews to explore barriers and facilitators. We assessed all written and verbal comments on the preliminary recommendations and revised the document accordingly. A major learning of the review process was that the presentation of the recommendations required more clarity since reviewers were familiar with their national context, but the recommendations were intended to be applicable in a more general manner. The implementation of some novel policies was considered challenging by some reviewers, and the relevance of accompanying measures was also stressed.

## 3 Results

Uptake of CA-ARTI POCTs in general practice may be limited for several reasons. Some of them, including funding perceived as insufficient by physicians or suppliers, or limited affordability for patients, might be addressed by fit-for-purpose funding and pricing policies. We developed seven recommendations for policy interventions in the areas of funding and pricing, the implementation of which is expected to contribute to enhanced use of CA-ARTI POCTs. Four recommendations relate to funding, including policy options regarding remuneration for physicians and reimbursement of the POCTs; these recommendations (RF1 to RF4) are presented in Table1. Table 2 is dedicated to three recommendations regarding pricing and procurement (one recommendation on price regulation / RP1 and two recommendations related to public procurement / RP2 and RP3). Some policy measures may only be applicable in certain health settings, which is also indicated (see the third column in Table 1 and Table 2). It is key to develop and undertake accompanying measures which are supportive or even required to ensure effectiveness of the recommended policy interventions (see the fourth column in Table 1 and Table 2).

## 4 Discussion

All seven proposed recommendations aim to encourage the uptake of POCTs but each of them may have positive and negative implications for different stakeholder groups, as outlined in the following discussion.

### 4.1 Funding policies

At least two recommendations in the policy area of funding (the proposal for an adapted tariff scheme for physicians / RF1, and implementation of product-specific reimbursement for CA-ARTI POCTs / RF4) are likely to lead to an increase in public funding. Beneficiaries include the suppliers, who would gain larger market shares, as well as physicians in case of the first mentioned measure. In addition, product-specific reimbursement would benefit suppliers and patients, as this measure would reduce the risk for patients to suffer from financial burden of the out-of-pocket payments for the POCT purchase. It is known from other areas of healthcare that out-of-pocket payments may lead to situations in which patients decide to not use a health technology (or they may not visit a health professional or health care facility at all) [34], [54], [55], [56], [57], [58], [59], [60], [61]. To note that the product-specific reimbursement policy for POCTs would be an appropriate measure in health systems where patients pay out-of-pocket for the tests (i.e., health systems organised based on physicians in their private practice, with a contract with health insurers), whereas it would make no difference in health systems, in which tests are procured by the national (or regional) health services and are applied in primary care facilities for free.

When patients have to pay for a CA-ARTI POCT, there are important factors that may influence their decision to agree on its use. These include affordability (which is lower for poorer people and vulnerable groups) and price elasticity. The latter measures the responsiveness of patients to changes in the price of a product (i.e., in case of high price elasticity, a small change in price will result in a large change in the demand for that product). While price elasticity is, in principle, low in health care (i.e., patients pay a given, even high, price to gain access to health technologies), it was found to differ depending on the type of product or service and patient group [62]. Further factors include overall low health literacy of patients, in particular related to antibiotics and AMR (including the expectation of several patients to receive an antibiotic prescription [63], [64]) and limited time resources and capacity of physicians to inform patients about appropriate antibiotic use and the role of diagnostics. A combination of these determinants may be responsible for a situation in which patients aim to avoid use of a POCT, for which they would have to pay, and prefer an antibiotic prescription, whose expenses are borne, at least partially, by the health system in most European countries [40], [65].

Our recommended funding measures add to other policies suggested in the literature and policy debate, which aim to tackle AMR by incentivizing the development of novel antibiotics, e.g., through well-equipped funds [5], [66], [67]. Those proposals are based on the rationale that addressing AMR comes at a cost, and this trade-off needs to be balanced when implementing the funding policy recommendations RF1 and RF4. However, solely increasing funding may not necessarily enhance use. Overall, it is important to design the measures carefully and appropriately to avoid unintended consequences, such as overuse of the POCTs [43], and to evaluate their performance. To strengthen the effectiveness of policies, conditionalities to public funding could be attached, as two of the other recommendations on funding (RF2 and RF3) propose.

For the time being, the pharmaceutical and the medical device sectors are organised separately, and competencies frequently sit with different institutions [44]. Recommendation RF2 aims to overcome this fragmentation, considering the role of CA-ARTI POCTs as companion and complementary diagnostics for antibiotics [68]. For companion diagnostics for precision medicine (e.g., in cancer care), cross-sectorial funding approaches have been implemented in a few European countries (e.g., Belgium, Germany) where a joint procedure for the inclusion of the medicines and their respective companion diagnostics in the outpatient benefits package scheme was established [69]. These examples serve as promising good practices whose transferability to antibiotics and CA-ARTI-POCTs could be explored but require breaking up the silos between the different sectors (pharmaceuticals and medical devices).

Similarly, making public funding of CA-ARTI POCTs dependent on data generation (RF3) would also constitute a novelty for AMR health products. While managed-entry agreements (MEAs) are frequently used for medicines with high price tags and also high uncertainty (e.g., gene therapies, cancer medicines), they are rather unknown for medical devices. An exemption is use of Coverage with Evidence Development (CED) for some diagnostics (but not CA-ARTI POCTs) in France, with the aim to gain more knowledge on the devices in practice [69], [70]. Evidence generation as basis for the improvement of the tests in terms of accuracy (measured by sensitivity and specificity) and consistency of the results in different populations could be one objective of an MEA, together with reduction of uncertainty for public payers. New data would support payers in their decision on whether, or not, to continue reimbursement of a CA-ARTI POCT, and, if yes, whether they would pay the same, or an adjusted reimbursement amount. MEAs in the pharmaceutical area may offer interesting lessons. An important learning is the necessity of clear definitions regarding the scope of data to be collected, responsibilities and subsequent consideration and use of the generated data. MEAs for POCTs could focus on managing uncertainty (with subsequent adjustment of the amount publicly funded), but there is no need for confidential discounts, which are commonly used in MEAs for medicines. Proponents in favour of confidentiality argued that the discounts for medicines would grant flexibility to manufacturers in a policy landscape characterised by the widespread use of the external price referencing policy in European countries [33], [71], which considers the prices of the same medicine in reference countries for their price setting [47]. However, with the absence of external price referencing for CA-ARTI POCTs [42], this argument is meaningless. Moreover, as the practice of confidential discounts has caused information asymmetry and an imbalance between sellers and purchasers [33], it would be in the interest of policy-makers to provide clear, transparent and well-defined processes for a more level playing field between payers and suppliers.

### 4.2 Pricing policies

Further to funding policies, pricing policies also aim to improve the affordability of purchasers, who may include public authorities, health professionals or patients. Since CA-ARTI POCTs used in the outpatient sectors of European countries are not subject to price regulation [42], its introduction (recommendation RP1), with the selection of the most appropriate pricing policy, or combination of policies, for CA-ARTI POCTs could be a first step to address the potential access barrier of prices being perceived as too high (in relation to comparably low prices of “old” established antibiotics).

The plan of regulating prices is likely to be met with opposition, especially by suppliers who have been used to set the prices at their discretion. Thus, as part of preparation for the implementation of the policy, policy-makers are urged to have a conversation with the suppliers and communicate the rationale and possible benefits of the measure for suppliers (for instance, about the expectation of higher uptake of the tests). In addition, pricing policies could also be used as an incentive to reward higher quality of the POCTs (e.g., in terms of effectiveness, accuracy) by granting higher prices. Furthermore, as policy options are usually not intended to be stand-alone measures but implemented as part of a policy mix, possible drawbacks for suppliers from price regulation may be mitigated by other incentives, including innovative procurement models. We also propose subscription-based procurement arrangements (RP2), which offer predictability on expected volumes to suppliers.

There is some experience with subscription-based procurement models for health technologies. In the AMR area, subscription-based procurement arrangements were piloted for antibiotics [40], [65]. In England, a pilot of a fixed annual subscription fee (of 10 million GBP per product and year) was launched in 2019. Informed by experts, two medicines (cefiderocol and ceftazidime with avibactam) were selected for a HTA, for which the National Institute for Health and Clinical Excellence (NICE) developed a tailored methodology to facture the value of novel antibiotics [72]. Based on a positive opinion for both medicines, contracts were signed and took effect from July 2022 [73], [74]. In a pilot in Sweden, contracts with suppliers of reserve antibiotics were concluded, which guaranteed a fixed annual sales volume and offered additional payments in case of largely exceeding this threshold [75]. These subscription-based procurement models aim to compensate for the small size of the market, as it is intended to keep antibiotic use to the necessary level. Other models, including the Australian pioneer subscription-based procurement scheme for hepatitis C medicines, have been guided by a different thinking: Australia (and later also Louisiana and Washington in the United States) offered a fixed revenue to suppliers for treating an unlimited number of eligible patients with hepatitis C medications within a period of five years [76], [77]. Thus, the hepatitis C procurement models have served to secure access and use, while containing public budgets. This rationale of compensating suppliers for defined volumes would also apply to subscription-based procurements for CA-ARTI POCTs. Suppliers and public purchasers would know in advance the expected sales and public spending. For both parties, it would be crucial to have most accurate forecasts when they enter negotiation. In particular, robust predictions on needed POCTs over a longer period of time are necessary to avoid possible over-use of the diagnostics, in addition to clear guidance to doctors on when to use the test. It is also important to involve prescribers and encourage them to use the POCTs when applicable. While the idea of applying subscription-based procurement models to diagnostics (and not antibiotics) may appear unusual at first glance, but it can help increase predictability. In the literature, a similar lump sum model was proposed to prevent healthcare-associated infections caused by multidrug-resistant organisms in the hospital setting [78].

While the subscription-based procurement models are specific and their implementation and management may require large efforts and resources, there is also potential in optimising public procurement, which is commonly used in some European countries to purchase POCTs used in primary health units. The recommendation on more strategic procurement (RP3) aims to balance the trade-off between affordable and competitive prices and incentives for suppliers to market the POCTs. It is again the prospect of (guaranteed) volumes, continuity and predictability which are aimed to be achieved through practices of more strategic procurement, such as multiple-winner awards (so that more suppliers can serve the market simultaneously), granting more weight to other award criteria beyond the price (thus allowing procurers to indicate some of their expectations on the POCTs, e.g., with regard to quality, effectiveness), and offering large markets to suppliers through pooled procurement. These procurement procedures and techniques have also been promoted in the EU legislation (such as the EU Public Procurement Directive [79]).

### 4.3 Accompanying measures

Implementation considerations outlined in Table1 and Table2 are similar for most measures. This confirms the overall importance of accompanying measures and supportive action, such as an implementation plan, stakeholder engagement (e.g., with suppliers and prescribers), pilots in case of novel policies, as well as monitoring and evaluation (including investment into appropriate robust systems allowing to do so with acceptable workload for those involved).

Applying generation and appraisal of evidence as a guiding principle in decision-making, it is beneficial to collect and analyse data in the early stages as well as during use of the CA-ARTI POCTs in clinical practice. An extremely useful tool is HTA, for which methodological guidance, largely developed in the EUnetHTA project [80], is available. At the time of the study, HTA is not systematically used for funding and pricing decisions for diagnostics in European countries [42]. While the HTA methodology may need to be optimised for CA-ARTI POCTs to appropriately consider the specificities of these diagnostics, an HTA on a POCT to guide antibiotic prescribing, which the Irish Health Information and Quality Authority conducted [18], may serve as a model. Moving forward with strengthening funding and pricing policies for CA-ARTI POCTs, policy-makers are encouraged to take the opportunity to introduce HTA in an institutionalised manner.

While this article focused on *peri*-launch policy measures around funding and pricing, which have been an under-researched area and not in the attention of policy-makers, further policy action around POCTs is still needed, ranging from clear target product profiles, investment in research and development (including public funding), strong guidance for prescribers, educational measures for health professionals, information targeting the public to raise awareness about AMR and improve knowledge and acceptability of testing, and robust monitoring and evaluation frameworks. Funding and pricing policies are thus –though important– elements of a broader implementation strategy for POCTs.

## 5 Limitations

The research has some limitations. The development of the policy recommendations was based on qualitative research, which was country- and context-specific. Given the comparatively low level of policy implementation in this area, it was challenging to identify experts who could provide evidence to inform the policy recommendations. With regard to the review process, we acknowledge that, while we had a broad review process addressing several stakeholders, not all potential reviewers provided comments.

While a recommended policy measure would be, in principle, applicable in a health setting of defined characteristics, as specified, its implementation may still not feasible or appropriate at the current situation in a given European country given country-specific details in the existing health system and policy context. In addition, immediate introduction of some measures with major impacts (e.g., on public funding, on organisation and processes) may be challenging and would require several preparatory steps before actual implementation.

The presented policy recommendations aim to enhance use of POCTs by physicians in the community. Depending on the country context, use of POCTs by other outpatient health professionals, such as community pharmacists and community nurses, and in the inpatient setting, could also be encouraged, and for doing so, other policies would need to be developed.

## 6 Conclusions

AMR is a multi-faceted problem, and thus a range of actions in different areas are needed to address this global public health threat. Encouraging use of diagnostic tests is one among several policy options.

While different approaches to enhance the use of POCTs exist, little attention has been paid so far to the potential of funding and pricing policies to provide leverage. This may be attributable to the current situation in Europe, as no country has implemented price regulation for CA-ARTI POCTs and only a few countries include these diagnostics in an outpatient reimbursement list. In response, policy interventions recommended in this paper comprise some basic funding and pricing policies, such as the establishment of a reimbursement list for product-specific funding of eligible cost-effective POCTs and price regulation. In addition, novel policy measures were proposed that take into account the need of future-proof solutions. Some of the policy measures are only applicable in certain health settings, but where applicable, they allow taking policy action whose implementation, though challenging, does not require an overhaul of the existing system.

As known from other policy areas, it would be beneficial to implement a mix of policies which may be inter-connected, such as introduction of price regulation for those POCTs that are publicly funded.

The research also highlighted the need for breaking up silos and considering the introduction of policies that bridge between the medical device and pharmaceutical sectors given the strong linkage between POCTs and antibiotics.

When moving forward in policy implementation, supportive action is highly encouraged. Accompanying measures include systematic use of HTA as basis for funding and pricing decisions, stakeholder dialogue and patient engagement, as well as monitoring and evaluation. In cases where the intended objectives of a policy are not achieved or the occurrence of unwanted effects, e.g., an overuse of the POCTs, a process should be in place to allow adapting the policy. Finally, implementation of funding and pricing policies to encourage the uptake of POCTs would need to be supplemented by further policy action around POCTs in the *pre*-launch (e.g., research and development) and post-launch phases (e.g., educational measures).

## CRediT authorship contribution statement

**Sabine Vogler:** Writing – original draft, Visualization, Supervision, Resources, Investigation, Funding acquisition, Conceptualization. **Caroline Steigenberger:** Writing – review & editing, Project administration, Investigation, Conceptualization. **Friederike Windisch:** Writing – review & editing, Investigation, Conceptualization.

## Declaration of competing interest

The authors declare that they have no known competing financial interests or personal relationships that could have appeared to influence the work reported in this paper.
